# Moxibustion for abdominal pain in COVID-19: A protocol for systematic review and meta-analysis

**DOI:** 10.1097/MD.0000000000028596

**Published:** 2022-01-21

**Authors:** Xuhao Li, Tiantian Dong, Yi Hou, Zhibin Dong, Jiguo Yang

**Affiliations:** a Shandong University of Traditional Chinese Medicine, Jinan, Shandong, China; b Affiliated Hospital of Shandong University of Traditional Chinese Medicine, Jinan, Shandong, China.

**Keywords:** abdominal pain, coronavirus disease 2019, moxibustion, protocol

## Abstract

**Background:**

Coronavirus disease 2019 (COVID-19) is an acute respiratory infectious disease that makes breathing difficult and is often accompanied by abdominal pain and distension. Moxibustion, a special external treatment of traditional Chinese medicine, has shown beneficial effects in the treatment of abdominal pain. Currently, there is a lack of systematic reviews on moxibustion for the treatment of abdominal pain. We conduct this study to evaluate the efficacy and safety of moxibustion in the treatment of abdominal pain. This study is designed to evaluate the effectiveness and safety of moxibustion for abdominal pain in COVID-19.

**Methods:**

Randomized controlled trials from December 2019 to December 2021 will be included, without restrictions on language or publication date. PubMed, EMBASE, Cochrane Library, Web of Science, Chinese Biomedical Databases, China National Knowledge Infrastructure, Wanfang Database, and VIP Database were searched. Two researchers will independently select studies, extract data, and evaluate study quality. The Cochrane risk of bias tool for randomized trials will be used to assess the risk of bias in the included studies. Statistical analyses will be conducted using the RevMan 5.3 software.

**Results:**

This study aimed to prove the efficacy and safety of moxibustion for abdominal pain in patients with COVID-19. Our study provides a more accurate treatment method for abdominal pain during COVID-19. We will publish our results in a peer-reviewed journal.

**Conclusion:**

This study will provide more convincing evidence for clinicians to treat these conditions and help them make appropriate decisions.

**Ethics and dissemination:**

This study did not include personal information. Ethical approval was not required for this study.

**INPLASY registration number:**

INPLASY2021120104.

## 1 Introduction

Coronavirus disease 2019 (COVID-19), named by WHO, first broke out in Wuhan, Hubei Province, China, in late 2019.^[1,2]^ Caused by the coronavirus severe acute respiratory syndrome coronavirus 2, COVID-19 is generally susceptible.^[3]^ According to statistics from Johns Hopkins, as of June 30, 2020, 10,302,867cases of COVID-19 have been confirmed, and there have been over 505,517 deaths worldwide. The clinical manifestations and prognosis of COVID-19 are quite different. Most of the patients with mild symptoms (approximately 80.9%) have a favorable prognosis, with the main manifestations of fever, fatigue, and dry cough, and a few are accompanied by nasal congestion, runny nose, sore throat, and diarrhea. However, severe and critical patients (approximately 19.1%) have a poor prognosis. They have high fever after the onset of the disease, and dyspnea and hyponea occur 1 week later.^[4]^ COVID-19 has put considerable pressure on the worlds medical system and caused significant mortality and economic loss around the world. Therefore, the exploration of effective treatment has become a top priority. According to the latest report, abdominal pain is a common symptoms for delta variant cases.^[5]^ Nowadays, the treatment methods for abdominal pain of COVID-19 are mainly western medicines which may prolong viral replication in severe acute respiratory syndrome coronavirus 2 and might be associated with a worse COVID-19 clinical course.^[6]^ Therefore, alleviate the abdominal pain in COVID-19 patients is helpful to improve the quality of life.^[7]^

As one of the external treatment methods of traditional Chinese medicine, moxibustion has the functions of dredging meridians, promoting qi and blood circulation, channeling meridians, activating collaterals and relieving pai. Studies have proved that moxibustion has unique advantages in the treatment of abdominal pain and was widely used in worldwide.^[8]^ Stimulating acupoints can release endogenous opioid polypeptides from periaquittal gray matter, and activate opioid receptors in central nervous system to produce labor pains by simulating endogenous antipain substance enkephalin. During the COVID-19 epidemic, moxibustion has been used as a complementary treatment for COVID-19 in China and has been confirmed the efficacy of COVID-19 with routine regimens. According to published studies, moxibustion can effectively relieved the symptoms of abdominal pain in COVID-19, reduce the frequency of abdominal pain and shorten the time of abdominal pain. However, there is still a lack of high-quality evidence to support the effectiveness and safety of moxibustion on abdominal pain. Based on this, we designed this study to better understanding of the effectiveness and safety of moxibustion therapy for abdominal pain in COVID-19. There by paving the way for the future treatment of abdominal pain.

## 2 Methods

### 2.1 Study registration

Our protocol has been registered on the International Platform of Registered Systematic Review and MetaAnalysis Protocols (INPLASY). The registration number was INPLASY2021120104 (DOI: 10.37766/inplasy2021.12.0104).

We will follow the recommendations outlined in the Cochrane Handbook of Systematic Review of Interventions and the preferred reporting items for systematic reviews and meta-analysis protocols (PRISMA-P) statement guidelines. If amendments are required, we will update our protocol to include any changes in the entire research process.

### 2.2 Criteria for including studies

#### 2.2.1 Types of studies

Randomized controlled trials of moxibustion in the treatment of abdominal pain will be comprehensively searched without restrictions on language or publication date. Additionally, unpublished documents were manually searched. Excluding nonrandomized controlled trials, reviews, experimental studies, clinical case reports, and animal research literature.

### 2.3 Types of participants

Subjects with documented COVID-19 with abdominal pain lasting 2 weeks or longer. There were no restrictions on sex, education, race, or disease stage, aged between 18 and 65 years. Patients with a history of abdominal pain prior to COVID-19 infection were excluded.

### 2.4 Types of interventions and comparisons

In addition to the treatment of COVID-19, treatment group interventions comprised moxibustion, direct moxibustion, separated moxibustion, moxibustion therapy, warm moxibustion or indirect moxibustion, and other moxibustion methods. Patients in the control group will receive other treatment without moxibustion.

### 2.5 Types of outcomes

The following information was collected from each study: publication year, country, ethnicity, specimen source, sex and age, case and control numbers, primary outcomes, and safety outcomes.

#### 2.5.1 Primary outcomes

Clinical variables will be set as the primary outcomes, such as abdominal pain frequency, abdominal pain intensity, duration of abdominal pain, duration of use of painkillers, and quality of life.

#### 2.5.2 Safety outcomes

The incidence of adverse events.

### 2.6 Search strategy

The Cochrane Central of Controlled Trials, PubMed, EMBASE, Cochrane Library, Web of Science, Chinese Biomedical Databases, China National Knowledge Infrastructure, Wanfang Database, and VIP Database. Using different databases, we combined keywords and free words for a comprehensive search. The complete PubMed search strategy is shown in Table 1.

**Table 1:** PubMed search strategy.

Number	Search items
#1	“moxibustion” [Title/Abstract] or “direct moxibustion” [Title/Abstract] or “indirect moxibustion” [Title/Abstract] or “separated moxibustion” [Title/Abstract] or “moxibustion therapy” [Title/Abstract] or “warm acupuncture” [Title/Abstract]
#2	“COVID 19” [Title/Abstract] or “2019-nCoV” [Title/Abstract] or “coronavirus disease 19” [Title/Abstract] or “coronavirus disease 2019” [Title/Abstract] or “disease 2019 coronavirus” [Title/Abstract] or “sars coronavirus 2 infection” [Title/Abstract] or “SARS-CoV-2” [Title/Abstract]
#3	“abdominal pain” [Title/Abstract] or “abdomen” [Title/Abstract] or “Epigastric pain” [Title/Abstract] or “Lower abdominal pain” [Title/Abstract] or “stomachache” [Title/Abstract] or “bellyache” [Title/Abstract] or “celialgia” [Title/Abstract] or “mulligrubs” [Title/Abstract]
#4	“Randomized controlled trial” [Title/Abstract] or “Controlled clinical trial” [Title/Abstract] or “clinical tria lrandomized” [Title/Abstract]
#5	#1 and #2 and #3 and #4

### 2.7 Data collection and analysis

#### 2.7.1 Selection of studies

**Figure 1:**
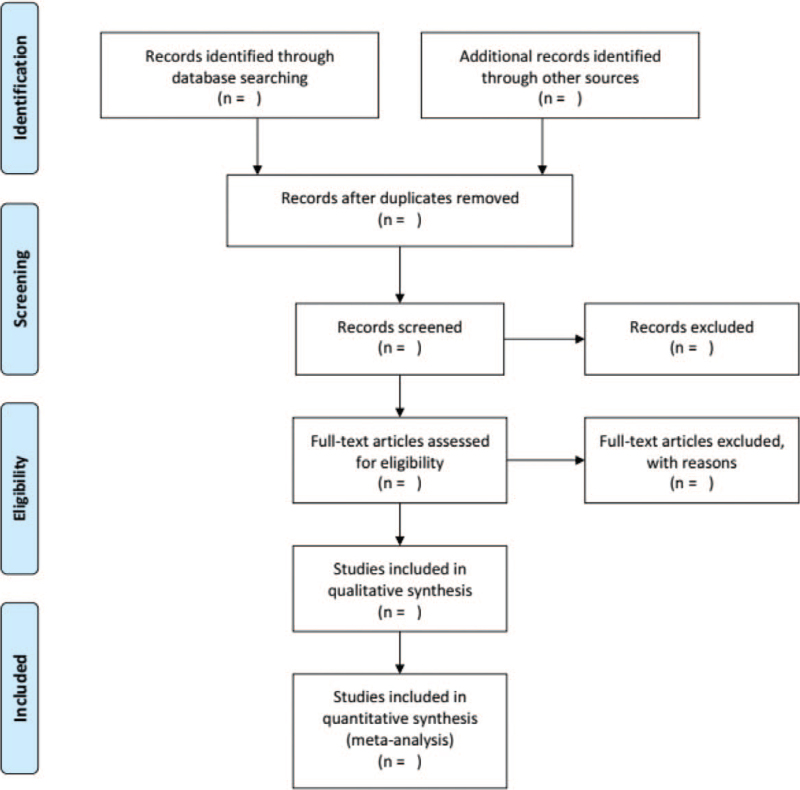
The PRISMA flow diagram.

The search strategy and study selection were independently performed by 2 researchers (XHL and TTD) in a database search, and the final research choices were agreed upon. Two researchers independently evaluated the same article to determine their eligibility for inclusion and resolved differences through consensus. Further unresolved discrepancy was managed by a third reviewer (YH). The selection process was summarized using a PRISMA flow diagram. The details of the selection procedure for the studies are shown in the PRISMA flow chart (Fig. 1).

#### 2.7.2 Data extraction and management

Two researchers (XHL and TTD) from the database for data extraction and data management, including the basic information of the included studies, baseline characteristics of the subjects, intervention and control measures, key elements of bias risk assessment, and outcome indicators. If there is any disagreement between the 2 researchers during the data extraction process, the panel jointly arbitrates and makes a decision.

#### 2.7.3 Dealing with missing data

If the information is missing or unclear, we will try to contact the corresponding author for more detailed information. If it fails, we analyze it based on the available data.

#### 2.7.4 Assessment of risk of bias

In this study, the Cochrane Handbook for Systematic Reviews of Interventions version 6 will be used to assess a broad category of biases. Two researchers will determine bias based on the following items: random sequence generation, allocation concealment, blinding of the participants and personnel, blinding of the outcome assessments, incomplete outcome data, selective reporting, and other sources of bias. Studies will be evaluated as “low-risk, high-risk,” or “clear risk.” Inconsistencies were resolved by discussion with other reviewers (ZBD).

#### 2.7.5 Assessment of quality of evidence

All studies used the Newcastle–Ottawa Scale assessment scale^[9]^ for evaluation. The evidence quality will be evaluated by 2 viewers (XHL and TTD) independently with the grading of recommendations assessment, development, and evaluation. The scale includes 9 items, covering 3 dimensions. The study was awarded 1 point for each item. The Newcastle–Ottawa Scale scores range from 0 to 9, with higher scores indicating better quality. In this study, a score of ≥6 was considered to be of better quality.

#### 2.7.6 Measures of treatment effect

RevMan 5.3 software (Cochrane Collaboration, Nordic Cochrane Center, Copenhagen, Denmark) was used to conduct this meta-analysis. For continuous results, the data were expressed as the mean difference or standard mean difference with a 95% confidence interval. When dichotomous data were available, a hazard ratio with 95% confidence interval was used. When binary data exist, the RR format is changed for the analysis.

#### 2.7.7 Heterogeneity evaluation

Clinical heterogeneity and statistical heterogeneity between the studies were assessed. Clinical heterogeneity was judged according to the similarity of research objects, intervention measures, control, and outcome indicators, and statistical heterogeneity was evaluated by *I*^*2*^. If *I*^*2*^ ≤ 50% and *P* > .1, statistical homogeneity was considered to be good. A fixed-effects model was used for merging. If *I*^*2*^ > 50% or *P* ≤ .1, the statistical heterogeneity was large, and the source of heterogeneity was further analyzed. After obvious clinical heterogeneity was excluded, a random-effects model was used for meta-analysis. When there is obvious clinical heterogeneity, it should be treated by subgroup analysis, sensitivity analysis, or only descriptive analysis.

#### 2.7.8 Assessment of reporting bias

When outcomes included more than 10 studies, we used Stata 14.0 (Stata Corp., College Station, Texas, USA) to assess the reporting bias using funnel plot and Egger test.^[10]^

#### 2.7.9 Data synthesis

We took advantage of RevMan 5.3 software for data analysis and synthesis. If there was no statistical heterogeneity between the results, a fixed-effects model was used. If there was statistical heterogeneity between the results, a random-effects model was used. If there was significant clinical heterogeneity, subgroup or sensitivity analysis was performed.

#### 2.7.10 Subgroup analysis

If feasible, we will conduct subgroup analysis based on different interventions, controls, treatment duration, and outcome indicators.

#### 2.7.11 Sensitivity analysis

We carried out a sensitivity analysis to investigate the robustness of the conclusions. The principal decision nodes include the method quality, sample size, and impact of missing data. Therefore, the impact of low-quality research on overall results was assessed.

#### 2.7.12 Ethics and dissemination

Since this study did not involve patient privacy, ethical approval was not required. Our research results will be shared and presented through conference reports and peer-reviewed journals.

## 3 Discussion

This study aimed to evaluate the efficacy and safety of moxibustion for the treatment of abdominal pain in COVID-19. Abdominal pain belongs to the category of “pain syndrome” in traditional Chinese medicine, and as an external treatment method of traditional Chinese medicine, moxibustion has the characteristics of simple and simple verification. It can not only prevent the occurrence of diseases but can also be used as an auxiliary treatment after the occurrence of diseases.

This study provides a new choice for a variety of treatment options for abdominal pain in COVID-19. We hope that this review will provide more convincing evidence for clinicians to treat these conditions and help them make appropriate decisions.

## Author contributions

All authors had access to the data and a role in writing the manuscript. All authors read and approved the final manuscript.

**Data curation:** Xuhao Li.

**Formal analysis:** Tiantian Dong.

**Methodology:** Xuhao Li, Tiantian Dong, Zhibin Dong, Yi Hou.

**Project administration:** Jiguo Yang.

**Resources:** Zhibing Dong.

**Software:** Xuhao Li, Yi Hou.

**Visualization:** Yi Hou.

**Writing – original draft:** Xuhao Li, Jiguo Yang.

**Writing – review & editing:** Jiguo Yang.
